# Prevalence of CMV infection among staff in a metropolitan children’s hospital – occupational health screening findings

**DOI:** 10.3205/dgkh000280

**Published:** 2016-09-27

**Authors:** Johanna Stranzinger, Jutta Kindel, Melanie Henning, Dana Wendeler, Albert Nienhaus

**Affiliations:** 1 BGW, Basics of Prevention and Rehabilitation, Occupational Medicine and Health Sciences Division, Hamburg, Germany; 2 Practising Specialist in Occupational Medicine, Hamburg, Germany; 3 Center of Excellence for Epidemiology and Health Services Research for Healthcare Professionals (CVcare), University Medical Center Hamburg-Eppendorf (UKE), Hamburg, Germany

**Keywords:** cytomegalovirus seroprevalence, staff in children's hospitals, risk factors CMV infection

## Abstract

**Background:** Staff in children’s hospitals may run an increased risk of cytomegalovirus (CMV) contact infection leading to a congenital CMV fetopathy during pregnancy. The main risk factor is close contact with inapparent carriers of CMV among infants (<3 years). We therefore examined CMV seroprevalence (SP) and possible risk factors for CMV infection among staff at a children’s hospital.

**Method:** In 2014, staff at a metropolitan children’s hospital were offered a CMV antibody test in the context of occupational health screening. Besides of anti-CMV immunoglobulin G (anti-CMV IgG) gender, age, profession, number of children and migration background were assessed and used as independent variables in multiple logistic regression. Women without a migration background (MIG) were considered as a separate group.

**Results:** The study included 219 employees. Women showed a significant higher risk than men of being CMV-positive (adjusted odds ratio [aOR] 3.0; 95% CI 1.1–7.8). The risk among age groups of 30 and over was double that of the under-30s (aOR 2.0; 95% CI 1.0–3.9); among those aged 40-plus it was aOR 2.3 (95% CI 1.1–4.7). Staff with an MIG tested more often positive than those without an MIG (95.5% versus 45.7%). CMV SP was 47.7% among women without an MIG. In this subgroup the probability of CMV infection increased with age (p=0.08) as well.

**Conclusion:** In the staff group as a whole there was a significant correlation between CMV SP, country of origin and age. We found no significant differences between occupational groups; perhaps our random sample was too small. Given the low CMV SP particularly in those without MIG, women who want to have children in particular must be protected from CMV infection. Follow-up studies should be undertaken to test whether good workplace hygiene offers sufficient protection for pregnant women and could be an alternative to prohibiting certain activities.

## Zusammenfassung

**Hintergrund:** Das Pflegepersonal in Kinderkrankenhäusern hat möglicherweise ein erhöhtes Risiko für CMV-Infektionen. Der Hauptrisikofaktor ist ein ungeschützter Kontakt zu unerkannten CMV-Ausscheidern insbesonders zu Kindern unter drei Jahren. Aus diesem Grund untersuchten wir die Seroprävalenz (SP) und mögliche Risikofaktoren der CMV-Infektion für Personal in Kinderkrankenhäusern.

**Methode:** Im Jahr 2014 wurde den Beschäftigten eines großstädtischen Kinderkrankenhauses bei der arbeitsmedizinischen Vorsorge ein Screening auf CMV-Antikörper angeboten. Außer dem CMV-Serostatus (Anti CMV IgG) wurden das Geschlecht, Alter, die Anzahl der Kinder und der Migrationshintergrund (MIG) erhoben und als unabhängige Variablen in die logistische Regression einbezogen. Frauen ohne MIG wurden als eigene Gruppe untersucht.

**Ergebnisse:** Die Studie umfasste insgesamt 219 Beschäftigte. Frauen hatten ein signifikant höheres CMV-Infektions-Risiko als Männer (adjustiertes Odds ratio [aOR] 3,0; 95% CI 1,1–7,8). Ab dem 30. Lebensjahr war das Risiko doppelt so hoch wie für die jüngeren unter 30 Jahren (aOR 2,0; 95% CI 1,0–3,9); diejenigen über 40 hatten ein aOR von 2,3 (95% CI 1,1–4,7). Beschäftigte mit Migrationshintergrund (MIG) waren häufiger CMV-seropositiv als diejenigen ohne MIG (95,5% versus 45,7%). Bei den letzteren erhöhte sich die Wahrscheinlichkeit für eine CMV-Infektion mit dem Alter (p=0,08).

**Schlussfolgerung:** Über alle Beschäftigten hinweg gab es einen signifikanten Zusammenhang zwischen CMV- Seroprävalenz, Migrationsgeschichte und Alter. Wir fanden keine signifikanten Unterschiede zwischen den einzelnen Berufsgruppen; möglicherweise war dazu die Stichprobe zu klein. In Hinsicht auf die niedrige CMV-Seroprävalenz der Frauen ohne MIG im gebärfähigen Alter müssen Schutzmaßnahmen besonders in der Schwangerschaft beachtet werden. In weiteren Studien sollte untersucht werden, ob eine verbesserte Hygiene am Arbeitsplatz Beschäftigungsverbote in der Schwangerschaft ersetzten oder reduzieren kann.

## Background

Cytomegalovirus (CMV) belongs to the herpes family of viruses (HHV 5). The majority of infections take an inapparent course, but typically lead to persistence of the virus. Among blood donors (age 18 to 60), an average seroprevalence (SP) of 45.8% is found. Depending on age and gender, this rises to >80 to 90% among the over-65s. Annual incidence among adult blood donors is highest among 30- to 35-year-olds (1.55%) [1]. The rate of seropositive individuals has declined continuously over the past two decades. This trend was observed in various population groups independently of socio-economic status (SES) [2], [3], [4], [5], [6]. 

Symptoms occur in 20% to 30% of infected persons. They are often non-specific, influenza-type symptoms. First and foremost among the groups with a high risk of CMV infections are individuals with a weakened immune system, in whom complex disease patterns may be triggered, affecting diverse organs such as lungs, liver, bowel and eyes. A further risk group are seronegative pregnant women, in whom CMV primary infection can cause diaplacental intrauterine death, congenital abnormalities of the foetus, or births of babies who are small for their gestational age [7]. In 10% of children who are born alive but sick, they lead to disabilities that are already visible in the postnatal phase, while a further 10% develop symptoms such as hearing impairments only at a later date [8]. The incidence of congenital CMV infection among live births is estimated to be between 0.3% and 1.2% in Germany [9], whereas among newly born infants in the Netherlands and Austria it is only 0.09% and 0.21% respectively [10], [11]. The consequences of prenatal CMV infection are most pronounced in the case of primary infections, and the earlier in pregnancy they set in, the more serious they are. Postpartal infection via breastfeeding is a mayor infection path, but without known risk to healthy and mature neonates. 

Close contact with children below the age of three is considered to be the most important risk factor. Children can be inapparent CMV carriers. Over several months or years, they secrete large quantities of CMV in urine and saliva after having themselves been infected prenatally via the placenta or postnatally via breast milk or contact with other carriers, usually children. That is why, in line with current government recommendations, a ban on employment in paediatric medicine, depending on the specific area of work and activities, has to be examined [12], [13]. Since no vaccination is available, hygiene training is designed to help prevent CMV infections in groups of at-risk persons [14], [15], [16], [17], [18], [19]. Valloup-Fellous et al. [20] in a major prospective incidence study described the effect of hygiene advice (hand disinfection without wearing gloves) on seronegative pregnant women in France (n=2583; between 2005 and 2007; CMV SP 50.1%): without hygiene advice, CMV seroconversion was higher in the first trimester than during the later course of pregnancy (n=16; 0.42% vs. 0.19%). Primary CMV infection was diagnosed more frequently in mothers with small children (12:4). Picone et al. [21] in an almost identical collective pointed out occupational groups at risk. A large proportion of infected pregnant women were healthcare workers. Among 14 women affected, five were nurses and two were doctors. As a limiting factor, one must point out that the occupational composition of the group as a whole was not described and the relative proportion of study participants in the medical sector is therefore unknown. 

In a Danish cohort study of fetal abnormalities, children of healthcare workers had a minimally higher frequency of minor or major congenital abnormalities than the children of other occupational groups (ORa: 1.09, 95% CI: 1.00–1.18 or 1.11; 95% CI: 1.00–1.22). However, it is not known whether this increased risk, insofar as it actually exists, was caused by CMV infections or by other possible risk factors such as shift work, physically strenuous activities, ionising radiation or hazardous substances [22].

Statutory accident insurance in Germany cover includes disabilities or deformities in children as a result of intrauterine infection if the underlying infection in the mother has an occupational cause. Between 2006 and 2013, 17 cases of CMV infection were reported to accident insurers in Germany, and 11 (65%) were recognised as occupational diseases (personal communication). In addition, in the same period a further six pregnant women who had contact with infected children were reported to the Institute for Statutory Accident Insurance and Prevention in the Health and Welfare Services (BGW) without an infection being confirmed following our analysis of 20 files concerning CMV related claims. Sixteen (80%) of the 20 files examined were of hospital staff. Four of the 14 cases of infection were recognised as occupational diseases. One case was that of a female paediatrician with a primary infection during pregnancy, which, however, gave birth to a healthy child. Otherwise, a paediatric nurse in a neonatal intensive care ward, a childcare worker in early years development and an internal medicine practitioner were affected.

A current survey found the rate of CMV SP among pregnant nursery school teachers to be 55% [23]. Among female staff in the paediatric ward of a university hospital, the CMV SP was 49% [24]. Otherwise, no current data are available on CMV SP among workers in Germany who are occupationally at risk. That is why we examined the CMV SP and possible risk factors for CMV infection among staff at a children’s hospital.

## Method

The company doctor in charge of occupational health screening in 2014 offered staff of a children’s hospital a blood test including a test for CMV antibodies (CMV Anti-IgG). She supplemented the anonymized laboratory dataset with voluntary information on gender, own children, occupation and country of origin. Subjects born outside the EU were classed as migrants. The laboratory tests for anti-CMV IgG were performed using customary ELISA test systems in an industrial laboratory independently of the hospital. The anonymized dataset and survey results were then transferred to the study centre, where they were analysed with the statistics software SPSS 22.

Differences between groups were examined by contingency table analysis using Pearson’s chi square test. In the case of ordinal data, the chi square test was used to analysis trend. The significance level was set at p<0.05. We used logistic regression to calculate odds ratios (ORs) for possible risk factors for CMV seroprevalence. The model was formed by stepwise regression using the change criterion [25], i.e., variables were eliminated step by step depending on their p value and their influence on the other variables in the model.

## Results

A total of 219 subjects took part in the study (Table 1 (Tab. 1)), of which more than 85% were women. Around 20% had two or more children. 10% were migrants. Care workers were the largest occupational group, accounting for 41.6% of the total. Of the subjects, 50.7% were anti-CMV IgG positive. 

The biggest differences between groups were associated with origin (Table 2 (Tab. 2)). Of the migrants, 95.5% were anti-CMV IgG positive, as against 45.7% of those born in the EU. The probability of a positive test result increased with age. From age 40, the OR was 2.3% (95% CI 1.1–4.7). Women were more frequently positive than men (OR 3.0; 95% CI 1.1-7.8). The probability of being anti-CMV IgG positive increased with the number of children (non-adjusted OR 2.5; 95% CI 1.3–5.1, no table). After adjustment for age, gender and origin, this effect is no longer found (Table 2 (Tab. 2)). The highest prevalence of anti-CMV was among cleaning and service workers (66.7 %) and the lowest among school students and apprentices (40 %). Carers showed a moderate prevalence (49.5 %). Prevalence among doctors of both sexes was slightly higher at 54.0%. However, these differences were not statistically significant. After controlling for age, the OR for cleaning and service workers as compared with carers fell to 0.5 (95% CI 0.1–2.1). There was a statistically significant increase in OR for migrants. However, this was distorted because of the low cell occupancy in the anti-CMV IgG negative migrants (OR 24.5; 95% CI 3.1–191.8). 

In the subgroup of women without a migration background (MIG) (N=174), 47.7% were anti-CMV IgG positive. Here, the test for trend with age was statistically significant (p=0.08). However, after adjustment for number of children and work activity, the OR is not statistically significant (Table 3 (Tab. 3)). 

## Discussion

We examined CMV SP in diverse occupational groups at a children’s hospital. No differences between occupational groups were found. Migration and age were statistically significant influencing factors. Our findings do not indicate an occupation-related increased risk of CMV infection. 

Other authors also report significant correlations between CMV SP and MIG [6], [3]. This effect was confirmed in staff at the children’s hospital. When interpreting CMV SP data, in addition to origin, age, gender and number of offspring, socio-economic status (SES) should be considered. Assessing SES by social insurance status, in pregnant women who were privately insured, SP was lower than in individuals with statutory insurance or in social welfare recipients (33.7 vs. 46.9 vs. 91.8%; [3], [26], [2]. In the study of CMV SP among pregnant staff (average age 31.0) at Frankfurt University Hospital, this observation was confirmed. There was a significant difference between doctors and caring occupations across all departments (37.5 vs. 53.4%; p=0.01). SP for hospital doctors was comparable with those of hospital workers who have no contact with patients (37.5 vs. 35.1%). Female paediatric staff were more frequently seropositive than the collective as a whole (49.1% vs. 43.9%), but the difference was not statistically significant. In addition, in paediatrics no distinction could be drawn between doctors and care staff due to the small size of the overall group [24]. In our study we found no significant differences between occupational groups, which can be considered as a surrogate for SES. However, the generalizability of our results is limited due to the small sample size and the cross-sectional design.

Some studies conducted in recent decades have suggested a low CMV risk for staff in children’s hospitals [27], [28], [29], [30], [31]. However, Lepage et al. [32] described an increased CMV risk among paediatric care assistants as against employees with no contact with patients. (OR: 1.8; 95% CI 1.1–2.8). 

Summing up, our study findings confirm the known effects of country of origin, gender, age and number of offspring on CMV SP. We found no work activity-specific risks. Since CMV SP in employees without a MIG is less than 50%, there is a risk of primary infection during pregnancy. It is therefore necessary to examine the serostatus of young female employees with special risks in departments of neonatology, oncology and intensive care and instruct them especially about CMV risk and measures of hygiene [33]. There is no consensual agreement in which working conditions CMV infection can be reliably prevented, and therefor still a need for further studies.

## Notes

### Competing interests

The authors declare that they have no competing interests.

## Figures and Tables

**Table 1:**
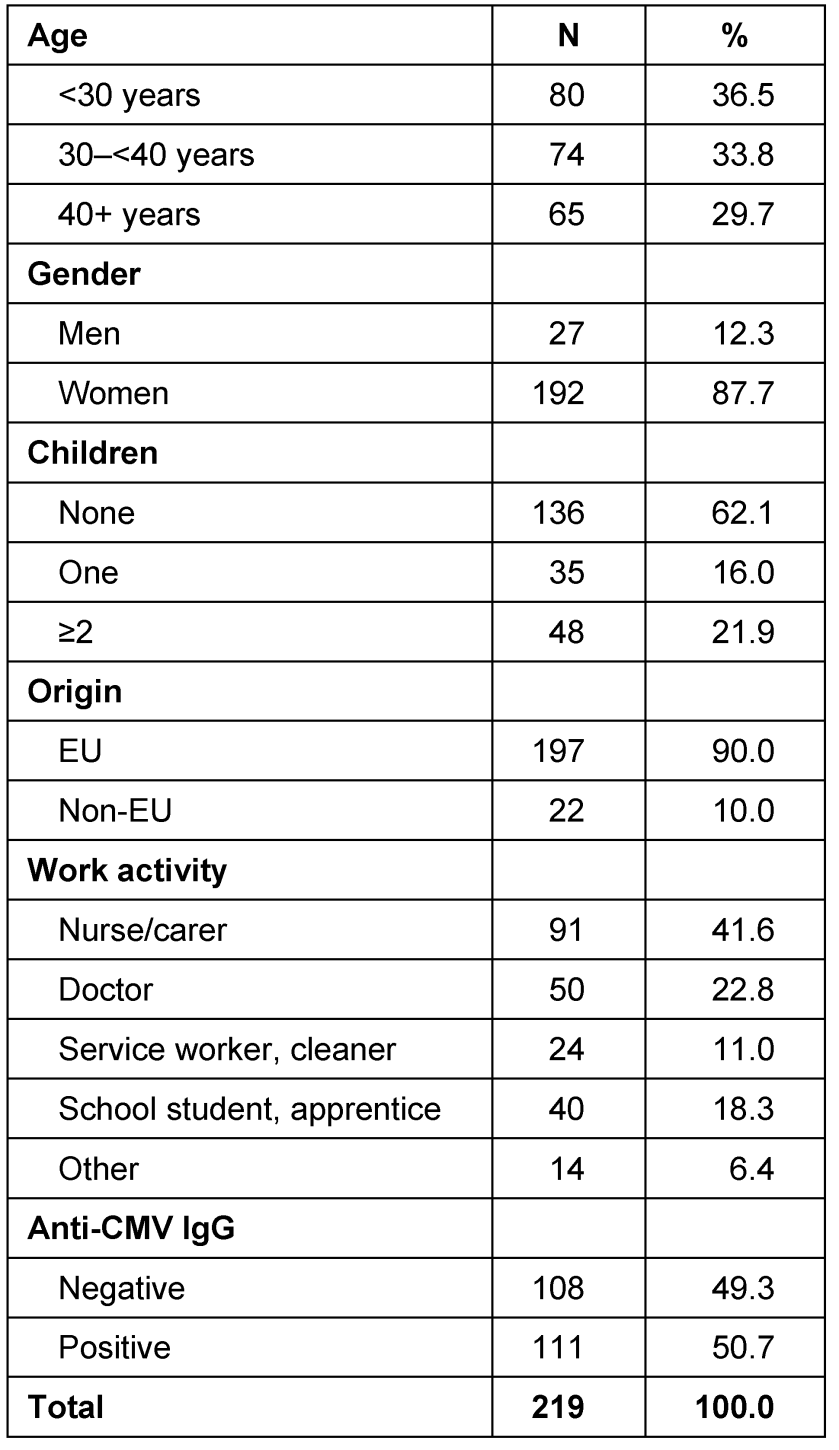
Description of population (N=219)

**Table 2:**
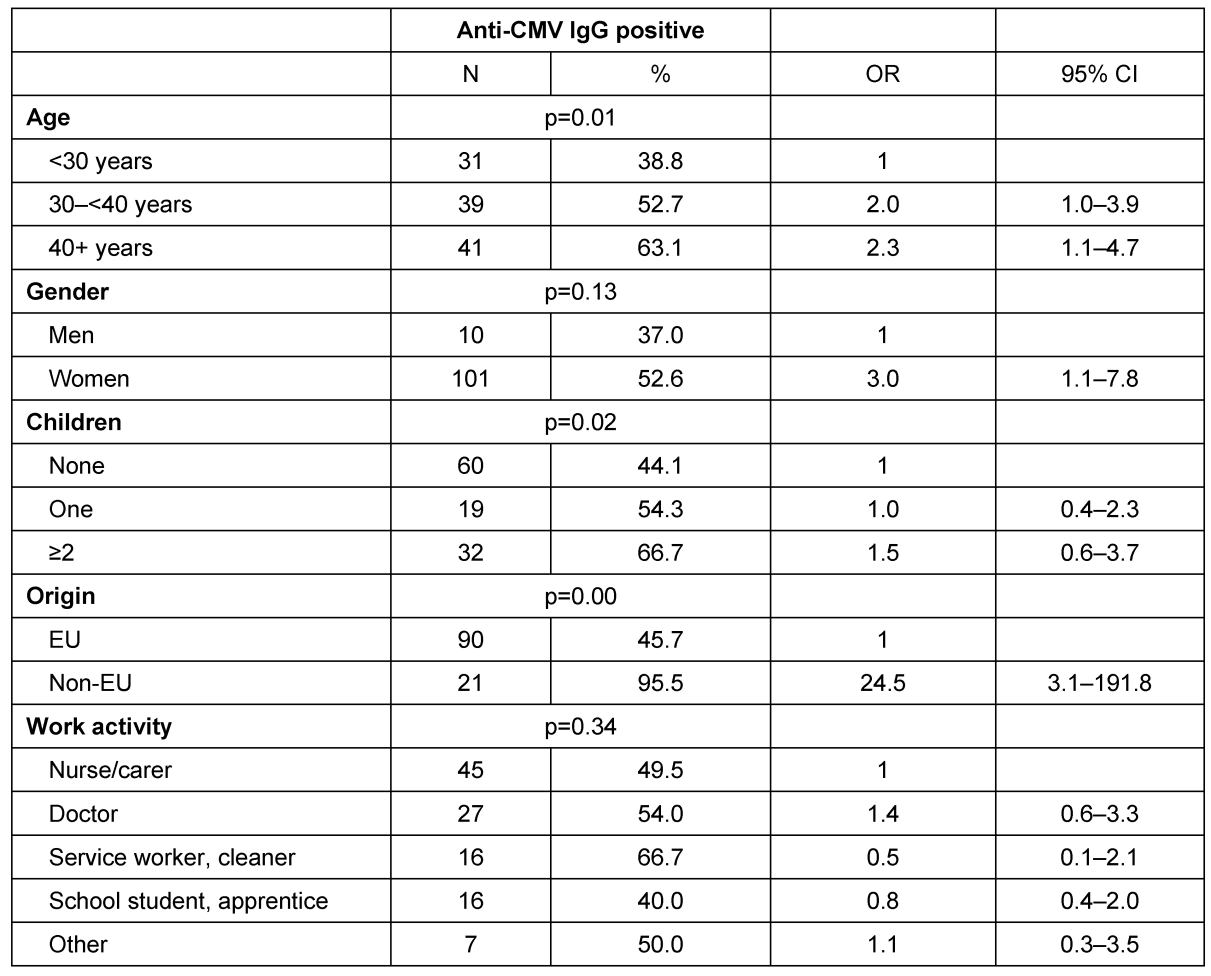
Number of persons with positive anti-CMV IgG (N=111) and OR adjusted for age, gender, number of children, origin and work activity

**Table 3:**
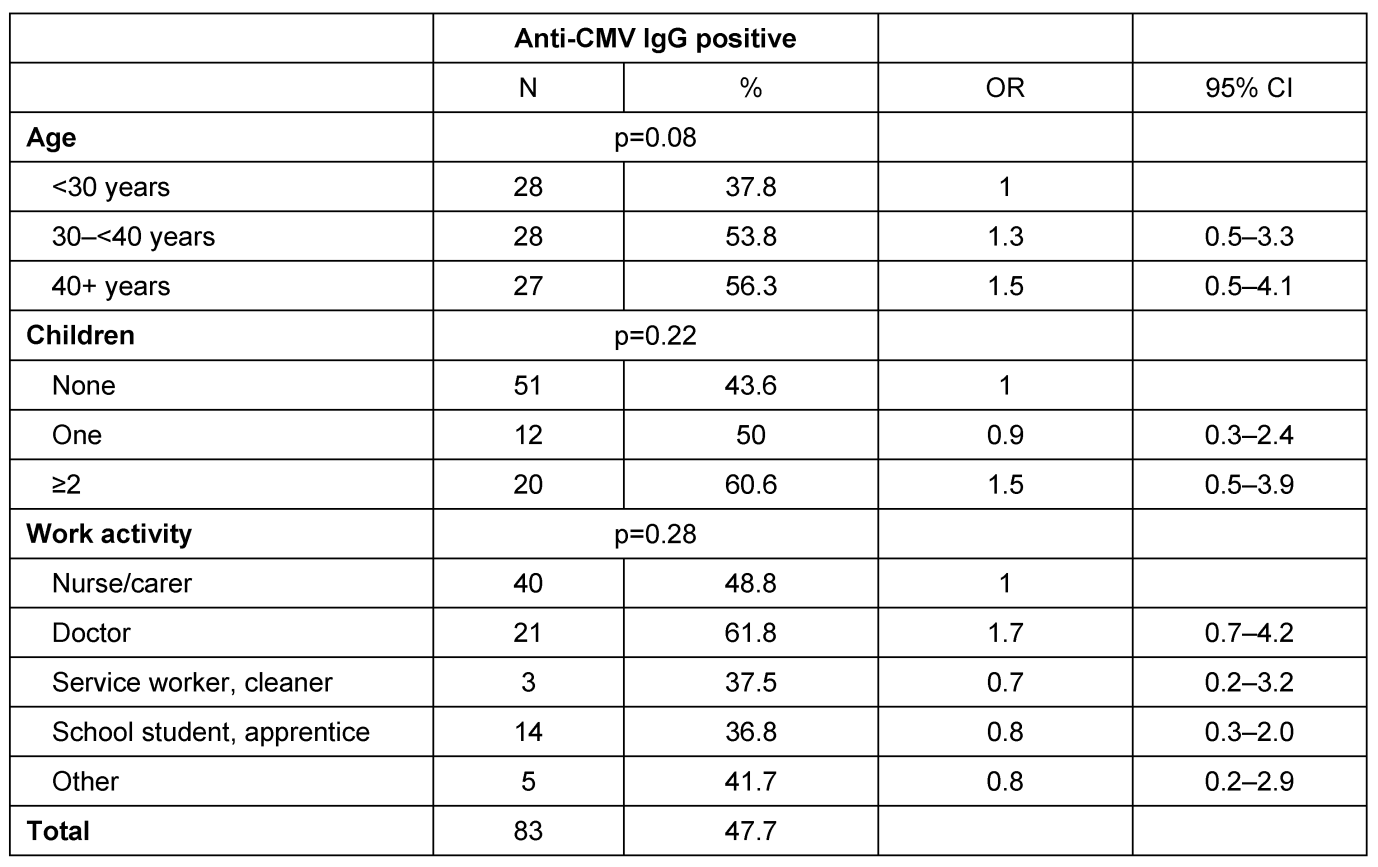
Number of women without an MIG with positive anti-CMV IgG (N=83) and adjusted OR
